# Vascular risk factors and progression of white matter hyperintensities in the Lothian Birth Cohort 1936

**DOI:** 10.1016/j.neurobiolaging.2016.03.011

**Published:** 2016-06

**Authors:** David Alexander Dickie, Stuart J. Ritchie, Simon R. Cox, Eleni Sakka, Natalie A. Royle, Benjamin S. Aribisala, Maria del C. Valdés Hernández, Susana Muñoz Maniega, Alison Pattie, Janie Corley, John M. Starr, Mark E. Bastin, Ian J. Deary, Joanna M. Wardlaw

**Affiliations:** a Brain Research Imaging Centre, The University of Edinburgh, Edinburgh, UK; b Neuroimaging Sciences, Centre for Clinical Brain Sciences, The University of Edinburgh, Edinburgh, UK; c Scottish Imaging Network, A Platform for Scientific Excellence (SINAPSE) Collaboration, Glasgow, United Kingodm; d Department of Psychology, The University of Edinburgh, Edinburgh, UK; e Centre for Cognitive Ageing and Cognitive Epidemiology, The University of Edinburgh, Edinburgh, UK; f Alzheimer Scotland Dementia Research Centre, The University of Edinburgh, Edinburgh, UK

**Keywords:** White matter damage, White matter hyperintensities, Vascular risk factors, Aging, MRI

## Abstract

We aimed to determine associations between multiple vascular risk factors (VRF) at ∼73 years and progression of white matter hyperintensities (WMH) from ∼73 years to ∼76 years. We calculated correlations and generalized estimating equation models of a comprehensive range of VRF at 73 years and change in WMH volume from 73 years to 76 years. Higher systolic (rho = 0.126, *p* = 0.009) and diastolic (rho = 0.120, *p* = 0.013) blood pressure at 73 years were significant predictors for greater WMH volume at 76 years in a simple correlation model. However, neither measured blood pressure nor self-reported hypertension at 73 years was significant predictors of WMH volume change in a fully adjusted model which accounted for initial WMH volume at 73 years. Lower high-density lipoprotein cholesterol (beta = −0.15 % intracranial, −1.80 mL; *p* < 0.05) and current smoking (beta = 0.43 % intracranial, 5.49 mL; *p* < 0.05) were the only significant VRF predictors of WMH volume change from 73 years to 76 years. A focus on smoking cessation and lipid lowering, not just antihypertensives, may lead to a reduction in WMH growth in the eighth decade of life.

## Graphical abstract

**Figure:**
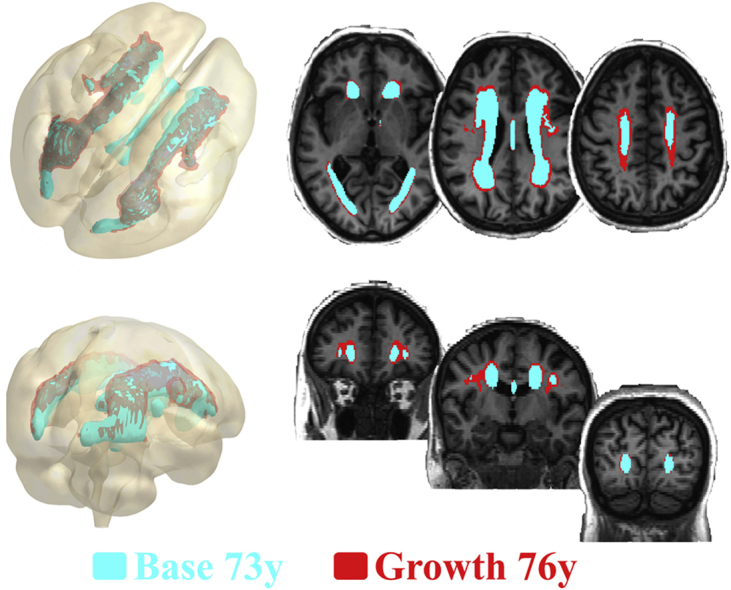

## 1 Introduction

Brain white matter hyperintensities (WMH) are common in community-dwelling older people and are associated with cognitive decline, dementia, stroke, and death (Brickman et al., 2012, Debette and Markus, 2010, Ritchie et al., 2015, Silbert et al., 2012, Valdés Hernández et al., 2013). It is therefore important to clarify the role of potentially modifiable targets, such as vascular risk factors (VRF; de Leeuw et al., 2002, Dufouil et al., 2001, Marcus et al., 2011, Murray et al., 2005), for intervention in progression of WMH.

Several cohort studies have assessed longitudinal WMH progression over approximately 2–6 years (Debette et al., 2011, Dufouil et al., 2001, Gottesman et al., 2010, Guo et al., 2009, Longstreth et al., 2005, Marcus et al., 2011, Sachdev et al., 2007, Schmidt et al., 1999, Schmidt et al., 2003, Schmidt et al., 2012, Taylor et al., 2003, van Dijk et al., 2008, Veldink et al., 1998, Wahlund et al., 1996). The strongest predictor of WMH progression appeared to be baseline WMH, where participants with the greatest volume of WMH at baseline had the greatest increases in WMH volume (Sachdev et al., 2007, Schmidt et al., 1999, Schmidt et al., 2003, Schmidt et al., 2007, Taylor et al., 2003, Veldink et al., 1998). These longitudinal studies mostly included participants across a wide age range and/or in midlife, assessed individual or few VRF (rather than combined VRF), used qualitative visual ratings for WMH progression, or did not measure baseline WMH (e.g., de Leeuw et al., 2002, Dufouil et al., 2001, Marcus et al., 2011).

The results of these previous studies conflict on the significance of VRF in progression of WMH. Some reported that hypertension and higher blood pressure were associated with greater increases in WMH volumes over time (e.g., de Leeuw et al., 2002, Debette et al., 2011, Dufouil et al., 2001, Gottesman et al., 2010, Guo et al., 2009, Longstreth et al., 2005, Marcus et al., 2011, Schmidt et al., 1999, van Dijk et al., 2008, Veldink et al., 1998), whereas others found that blood pressure was not associated with WMH progression (Sachdev et al., 2007, Schmidt et al., 2003, Taylor et al., 2003). The significance of smoking and cholesterol is also unclear from these prior studies (Debette et al., 2011, Sachdev et al., 2007, van Dijk et al., 2008), and one study found that no VRF were associated with WMH progression (Sachdev et al., 2007).

One reason for the conflict between previous reports may be their varying age ranges, and this suggests that different VRF may have differential effects on WMH throughout life. For example, the effect of blood pressure is strong in midlife (Debette et al., 2011) but apparently reduces after 70 years (van Dijk et al., 2008); whereas greater cross-sectional associations between cholesterol and WMH have been identified after 70 years (Ryu et al., 2014). Given that these potentially differential effects were identified in wide age ranges and cross-sectionally, it is important to clarify the significance of individual and combined VRF (Bangen et al., 2015, Maillard et al., 2015) in longitudinal WMH progression at specific ages. This is particularly true in the eighth decade of life where the risk of dementia increases substantially (Matthews et al., 2005).

Although birth cohorts may be limited in their generalizability by the range of environmental exposures represented, this study design allows the possibility to test the influence of individual and combined VRF associations in a large age-homogenous sample that mitigates against the strong effect of even small differences in age on WMH (Aribisala et al., 2014). None of the numerous previous studies that we have identified tested associations between WMH and a comprehensive range of VRF exclusively in the eighth decade of life.

With a homogenous sample of 439 community-dwelling participants from the Lothian Birth Cohort 1936 (LBC1936; Deary et al., 2007, Deary et al., 2012, Wardlaw et al., 2011), we aimed to determine the contribution of multiple VRF at ∼73 years to progression in WMH volume from ∼73 years to ∼76 years.

## 2 Material and methods

We followed “Strengthening the Reporting of Observational Studies in Epidemiology statement: guidelines for reporting observational studies” in preparation of this article (von Elm et al., 2007).

### 2.1 Standard protocol approvals, registrations, and patient consents

Approval for the LBC1936 study protocol was obtained from the Multi-Centre Research Ethics Committee for Scotland (MREC/01/0/56) and Lothian Research Ethics Committee (LREC/2003/2/29). All participants gave written, informed consent.

### 2.2 Participants

A total of 1091 (548, 50% male) community-dwelling participants in the Lothian area were recruited into the LBC1936 cohort study between 2004 and 2007 (Deary et al., 2007, Deary et al., 2012). Most participants completed the Scottish Mental Survey of 1947, in which almost all Scottish school children born in 1936 and attending school in June 1947 were cognitively tested. Of the 1091 participants initially recruited, 681 (362, 53% male) participants completed baseline brain magnetic resonance imaging (MRI) at mean age 72.7 years (standard deviation 0.7, range 71.1–74.3 years). Of the 681 participants with baseline brain MRI, 439 participants (n = 250, 55% male) returned for follow-up brain MRI at mean age 76.4 years (standard deviation 0.64, range 74.9–77.8 years) and had Mini Mental State Examination (MMSE) scores ≥24 at baseline and follow-up. We excluded subjects with possible dementia (MMSE<24) at either time point (n =1 2). It is the 439 participants with full brain imaging data and normal range MMSE at 73 and 76 years, which were used in the present analysis.

### 2.3 VRF assessment

According to previously published methods (Deary et al., 2007, Deary et al., 2012, Wardlaw et al., 2014), we measured several VRF during a Clinical Research Facility assessment at 73 years: systolic and diastolic blood pressure, total cholesterol, high-density lipoprotein (HDL) cholesterol ratio to total cholesterol (henceforth referred to as, “HDL cholesterol ratio”), and glycated hemoglobin (HbA1c). We used the average of 3 standing and 3 sitting systolic and diastolic blood pressure readings from an Omron 705IT monitor. We also included the after VRF by self-report: diagnosis of hypertension, hyperlipidemia, diabetes, history of cardiovascular disease, and smoking category (never, ex, and current).

### 2.4 Brain MRI acquisition and processing

Full brain MRI acquisition parameters were described previously (Wardlaw et al., 2011). Briefly, all participants had brain MRI on the same 1.5 T GE Signa Horizon HDx clinical scanner (General Electric, Milwaukee, WI, USA) maintained on a careful quality assurance program. The scanner and scanning protocol were the same at 73 and 76 years and acquired T1-, T2-, T2*-, and fluid attenuated inversion recovery-weighted images (Wardlaw et al., 2011).

We measured intracranial (ICV), whole brain, and WMH volumes in mL using a validated multispectral image processing method that combines T1-, T2-, T2*-, and fluid attenuated inversion recovery-weighted MRI sequences for segmentation (Valdés Hernández et al., 2010). All sequences were coregistered and tissue volumes estimated by cluster analysis of voxel intensities. According to STandards for ReportIng Vascular changes on nEuroimaging, we explicitly defined WMH as punctate, focal, or diffuse lesions in all subcortical regions (Wardlaw et al., 2013). WMH masks were manually edited by following STandards for ReportIng Vascular changes on nEuroimaging guidelines and using 3D-mask editing software, Multi-Image Analysis GUI (MANGO; http://ric.uthscsa.edu/mango/). Editing was overseen by a consultant neuroradiologist (Joanna M. Wardlaw). Cortical and subcortical infarcts were manually removed from WMH volumes during the editing process. We manually checked all segmented images for accuracy blinded to all clinical details, corrected errors, and excluded imaging-detected infarcts from WMH volumes (Wang et al., 2012).

### 2.5 Statistical analyses

#### 2.5.1 WMH volume distributions, changes and associations with VRF

Statistical analyses were performed in the Statistical Analysis System (SAS) version 9.4 (2002–2012 SAS Institute Inc.) and Matrix Laboratory (MATLAB) R2014a (1994–2014 The MathWorks, Inc.). Log transforming the positively skewed WMH distributions had little effect on our results, and therefore, we maintained their original scale (proportion of ICV) to simplify interpretation. We assessed changes in WMH and whole brain volumes from 73 years to 76 years using paired *t*-tests. We calculated Pearson correlations between volumes and Spearman correlations between VRF because a number of the VRF were categorical.

#### 2.5.2 Generalized estimating equations (GEEs) to model associations between VRF at 73 years and change in WMH from 73 years to 76 years

We used GEE, “PROC GENMOD” in SAS (SAS Institute Inc., 2010), to model associations between VRF at 73 years and change in WMH volume from 73 years to 76 years (Schmidt et al., 2003, Twisk, 2003). We controlled for overall head size in the first GEE model by dividing WMH volume by ICV and in a second model with raw WMH volume as the dependent variable and ICV included as an independent variable. We modeled smoking as a “dosage,” i.e., linear, variable (Karama et al., 2015), and as a categorical variable in 2 separate GEE. We adjusted GEE models for cardiovascular disease history, gender, and body mass index as in previous studies (Debette et al., 2011, Schmidt et al., 1999). We tested the variance inflation factor (VIF) of each independent variable using “PROC REG” in SAS to identify potential multicollinearity and volatile regression estimates. VIF ≥10 may indicate problematic multicollinearity (Belsley et al., 1980). Although systolic and diastolic blood pressures are highly correlated, we maintained both as separate independent variables (rather than pulse pressure) because their respective VIF were approximately 1.5 each. The VIF of low-density lipoprotein cholesterol was 13.2, and therefore, we only included HDL cholesterol ratio and total cholesterol in our GEE models. All other variables had VIF below 5.

## 3 Results

### 3.1 Changes in WMH and whole brain volume from 73 years to 76 years

**Fig. 1:**
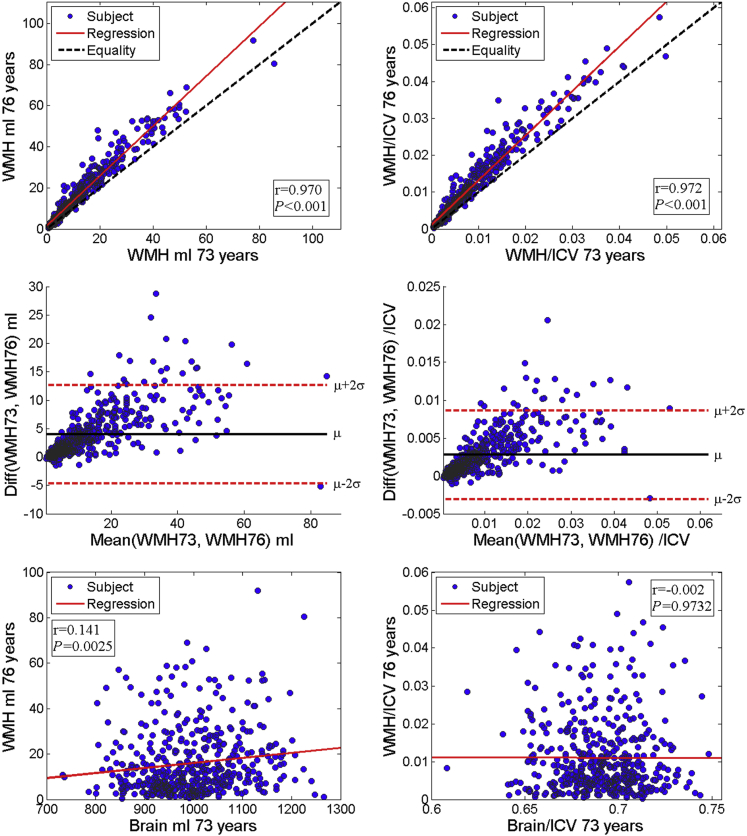
Plots of WMH and whole brain volume at 73 years and 76 years. Scatter plots of WMH volume at 73 years and WMH volume at 76 years (raw left top panel, ICV corrected right top panel); difference versus mean plots of WMH volume at 73 years and WMH volume at 76 years (raw left middle panel, ICV corrected right middle panel); and whole brain volume at 73 years and WMH volume at 76 years (raw left bottom panel, ICV corrected right bottom panel). Abbreviations: ICV, intracranial volume; WMH, white matter hyperintensities.

Mean changes in WMH and whole brain volume from 73 years to 76 years are summarized in Table 1 and Fig. 1. Whole brain volume generally decreased with age (paired Cohen's *d* of change = −0.95), and WMH volume generally increased with age (paired Cohen's *d* of change = 0.95).

**Fig. 2:**
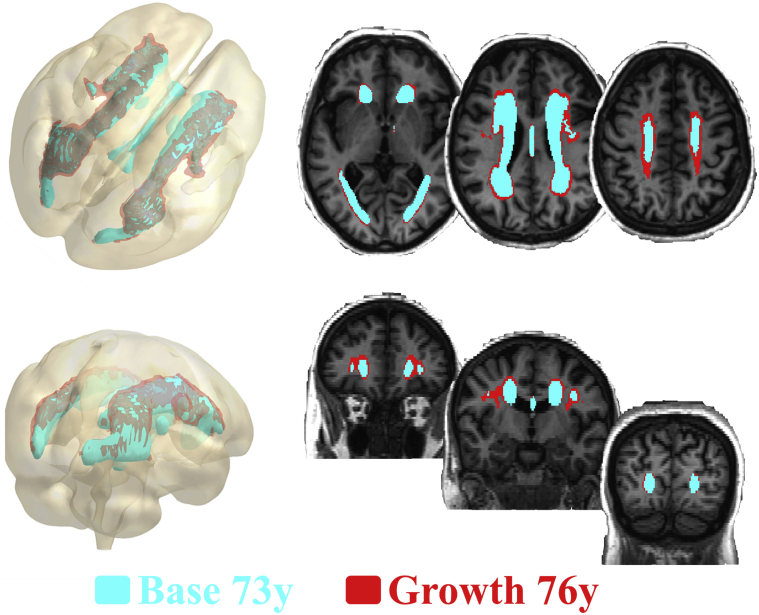
Growth of WMH volume from 73 years to 76 years. The blue volume shows where 95% of WMH occurred at 73 years. The red volume shows where 95% of the growth in WMH occurred from 73 years to 76 years. The beige volume is mean brain volume at 73 years. Abbreviation: WMH, white matter hyperintensities.

The mean WMH volume of 11.9 ± 11.7 mL at 73 years and 15.9±14.6 mL at 76 years (Table 1) indicates a relative mean increase of 33.6% over 3 years (4.0 mL ± 4.3 mL or 0.28% ± 0.29% of ICV). This growth of WMH was generally in the lateral superior direction, extending centrifugally from the ventricles and superiorly toward the vertex, with less growth posteriorly in the occipital poles (Fig. 2).

Fig. 1 shows that there was increased growth of WMH volume from 73 years to 76 years in those with bigger WMH volumes at age 73 years. There was a strong correlation between WMH volume at 73 years and 76 years (r = 0.97, *p* < 0.0001). Bigger raw WMH volumes at 76 years were associated with bigger raw whole brain volumes at 73 years (r = 0.14, *p* = 0.003), but ICV normalized whole brain volume at 73 years was not associated with ICV normalized WMH volume at 76 years (r = −0.002, *p* = 0.97).

Participants who did not return at 76 years had slightly smaller whole brain volumes at 73 years compared with participants who did return (68.5 %ICV vs. 69.1 %ICV; *t* = −2.98, *p* = 0.0030). However, participants who did not return at 76 years did not have bigger WMH volumes at 73 years (0.92 %ICV vs. 0.83 %ICV; *t* = 1.21, *p* = 0.2258).

### 3.2 Prevalence of VRF

The prevalence of VRF at 73 years did not significantly differ between participants who returned and those who did not return for follow-up (Table 2). The longitudinal changes in prevalence of VRF in returning subjects are in Table 3.

There were significant increases in the incidence of self-reported hypertension, hyperlipidemia, and history of cardiovascular disease; there was a significant average increase in HbA1c and decrease in total cholesterol from 73 years to 76 years (Table 3).

### 3.3 Spearman correlations between VRF at 73 years and WMH volume at 73 years and 76 years

There was a negative correlation between HDL cholesterol ratio at 73 years and WMH volume at 76 years (rho = −0.178, *p* < 0.001) and positive correlations with systolic (rho = 0.126, *p* = 0.009) and diastolic (rho = 0.120, *p* = 0.013) blood pressure at 73 years and WMH volume at 76 years. Associations between VRF at 73 years and WMH at 73 years were approximately equal to associations between VRF at 73 years and WMH at 76 years (Table 4). Smoking at 73 years was not a significant predictor of WMH at 76 years in this simple correlation model.

### 3.4 GEEs of associations between VRF at 73 years and WMH change from 73 years to 76 years

GEE of associations between VRF at 73 years and change in WMH volume from 73 years to 76 years are summarized in Table 5.

Lower HDL cholesterol and current smoking (entered as binary variable) were the only significant VRF predictors of increased WMH growth from 73 years to 76 years (Table 5). Smoking was not a significant predictor when considering it as a “dosage,” i.e., linear variable, in either the “%ICV” or “mL” models (beta = 0.1272 %ICV, 1.7120 mL; standard error = 0.0736 %ICV, 0.9875 mL; Z = 1.73, 1.73; *p* = 0.0840, 0.0830). Gender was a significant predictor of growth when raw WMH volumes were the dependent variables, and ICV was entered as a model predictor; with females having greater WMH growth than males (Table 5).

## 4 Discussion

We have found, from a comprehensive range of VRF, that lower HDL cholesterol ratio and current smoking at 73 years were associated with increased WMH volume progression from 73 years to 76 years. Higher diastolic and systolic blood pressure at 73 years appeared to be significant predictors of greater WMH volume at 76 years in a simple correlation model. However, neither measured blood pressure nor self-reported hypertension at 73 years were significant predictors of WMH volume change in a GEE model which accounted for initial WMH volume at 73 years. Females appeared to have increased rates of change in WMH when modeling raw WMH volumes as dependent variables and including ICV as an independent variable.

Previous studies of VRF and WMH have found that, rather than cholesterol and smoking, higher blood pressure is the greatest predictor of increased rates of WMH progression (e.g., de Leeuw et al., 2002, Debette et al., 2011, Gottesman et al., 2010, Guo et al., 2009, Longstreth et al., 2005, Marcus et al., 2011, Schmidt et al., 1999, van Dijk et al., 2008, Veldink et al., 1998), and there are a number of possible reasons for this discord. These studies generally had younger participants than studied here and/or had a wide age range of participants, included one or few VRF, did not account for baseline WMH volume, and/or used qualitative WMH ratings. Our results are consistent with the Rotterdam Scan Study of a wider age range that found the associations between blood pressure and WMH to significantly reduce after 70 years (van Dijk et al., 2008); the Austrian Stroke Prevention Study which found an association with hypertension to disappear when considering baseline WMH (Schmidt et al., 2003); a recent cross-sectional study that found higher cholesterol was associated with bigger WMH in subjects aged ≥70 years but not in younger subjects (Ryu et al., 2014); and with recent neutral results between blood pressure lowering and WMH volume in randomized clinical trials (Weber et al., 2012). Considering existing literature and the results from our study, there is increasing support for the theory that VRF have differing effects on WMH progression at different stages of life. Our results suggest that nonsmoking and higher HDL cholesterol levels are associated with less growth of WMH in the eighth decade of life, giving support to further age-specific trials of lipid lowering and lifestyle modification to prevent progressive vascular brain damage.

Smoking was not a significant predictor of WMH change when considered as a “dosage” (i.e., linear) variable in the GEE model compared with when considering it as a categorical variable. There is methodologic support for both approaches. The categorical approach may be favored because linear variables generally consist of continuous data (categories are not continuous). The dosage approach may be considered favorable in these data where only a small percentage (∼6%) of subjects were current smokers at 73 years, and results are based on unbalanced participant groups. However, in previous work with a more balanced sample of current and nonsmokers, current smoking was the strongest VRF associated with brain vascular damage when considered as a total burden of small vessel disease (Staals et al., 2014). This suggests that the categorical approach may be the most suitable one for assessing associations between smoking and vascular brain damage, but future work is required to confirm this.

Gender was not a significant predictor of WMH change when considering WMH as a proportion of ICV; however, females had significantly higher rates of change when raw WMH volumes were corrected by inclusion of ICV as an independent variable. Previous work in a multiple sclerosis–matched control study (n = 36) found that regression-based correction, rather than proportional correction, may have advantages for reducing parameter estimate errors (Sanfilipo et al., 2004). This methodologic concern has apparently received little else attention in the literature, but our regression-based result of increased progression of WMH in females is consistent with the higher number of strokes in females compared with males (American Stroke Association, 2014). However, further work is required to determine whether regression-based approaches do adequately account for ICV and whether females are indeed at increased risk of progression of WMH in the eighth decade of life.

WMH volume at baseline was the major determinant of WMH change and this is confirmatory of many previous reports (Sachdev et al., 2007, Schmidt et al., 1999, Schmidt et al., 2003, Schmidt et al., 2007, Taylor et al., 2003, Veldink et al., 1998). The relative mean increase in WMH volume from 73 years to 76 years of 33.6% (4.0 mL or 0.28% of ICV) equates to an 11.2% relative mean increase per year. This is approximately equal to the average rate of WMH volume progression in 3 previous studies of community-dwelling nondemented participants that found increases of 6.5% (Silbert et al., 2012), 13.35% (Taylor et al., 2003), and 13.2% (Sachdev et al., 2007) per year, at ages 55–102 years (Sachdev et al., 2007, Silbert et al., 2012, Taylor et al., 2003). The range of ages in these other studies may explain why the mean rate of WMH progression that we found was approximately equal to their combined average rate of progression (11.2% relative mean increase per year vs. 6.5%–13.35% per year). Furthermore, our finding that progression of WMH was least in the occipital poles is also consistent with a previous regional analysis (Sachdev et al., 2007).

Our study had a number of strengths. Whereas previous studies may have had younger and/or wide age groups where individual or few VRF were assessed, we concurrently assessed associations between a comprehensive range of VRF and progression of WMH exclusively in the eighth decade of life. This is an important decade of life where the risk for dementia increases substantially (Matthews et al., 2005). Our age-homogeneous sample of 439 community-dwelling older participants (assessed at 73 years and 76 years) provided increased statistical power to avoid a type II error (false negative) and correctly detect associations by having less need to statistically control for age (Freedman et al., 2007). Age is an extremely important confounding variable in WMH as we have previously found cross-sectional differences because of age even within the narrow range studied in LBC1936 (Aribisala et al., 2014). As well as the narrow age range, other novel features of the LBC1936 study (e.g., all participants are white Caucasian) may have minimized any potentially strong confounding effects that factors such as mixed ethnicity and geography might have had in a less homogeneous sample. An additional and important strength of our study is that we measured WMH volumes using a validated, multispectral, and quantitative technique that carefully excluded the effect of potentially confounding infarcts (Valdés Hernández et al., 2010, Wang et al., 2012, Wardlaw et al., 2011, Wardlaw et al., 2013). Finally, the raw brain MRI images from which we measured WMH volumes were obtained using the same quality controlled protocol on the same MRI scanner at both time points (Wardlaw et al., 2011).

Despite these strengths and consistency with previous work (e.g., Ryu et al., 2014, Sachdev et al., 2007, Schmidt et al., 2003, Taylor et al., 2003, van Dijk et al., 2008), our study had limitations. All but n = 25 subjects appeared to have an increase in WMH volume over time. We used a validated segmentation procedure and individually checked WMH masks for each subject at baseline and follow-up to minimize the effect of measurement error but some degree of error, e.g., due to motion and/or registration, will always be present in quantitative measurements of WMH. However, our data on WMH progression are consistent with previous studies of community-dwelling subjects that reported limited or no regression of WMH within measurement error (Sachdev et al., 2007, Schmidt et al., 1999, Schmidt et al., 2003, Veldink et al., 1998). We did not include duration of illness or self-reported use of VRF modifying treatments, e.g., statins or antihypertensive drugs, in the GEE but our inclusion of measured blood cholesterol, HbA1c, and blood pressure in the analysis will have accounted for the effectiveness of treatment to manage hypertension, diabetes, or hyperlipidemia. There were significant increases in the incidence of self-reported hypertension, hyperlipidemia, history of cardiovascular disease, and HbA1c increased on average from 73 years to 76 years. This suggests that any VRF associations with WMH could be accounted for by a general worsening of health. However, many of the measured VRF were stable over time and overall participants were still active and healthy enough to function in the community at 76 years. Participants who returned at 76 years had slightly bigger brain volumes at 73 years compared with participants who did not return, and therefore, the remaining participants that were used in this analysis may be slightly biased toward healthier community-dwelling participants. However, returning participants did not have smaller WMH volumes, our primary outcome measure, at 73 years. Mean follow-up time was 3.7 years, and this may not be long enough to capture large-enough changes to detect the small influences of risk factors (Johnson et al., 2012). However, follow-up times of approximately 3 years are common in studies and trials of WMH progression (Brickman et al., 2012, Schmidt et al., 1999, Schmidt et al., 2012, van Dijk et al., 2008), and we are assessing the same participants 6 years after baseline. These motivated participants score well on cognitive tests and broader generalizations from this sample may be limited in other populations. The narrow age range of LBC1936 precludes generalization of these results to other age groups, but our results are not intended to be generalized to other age ranges where VRF likely have a different impact on WMH progression (Debette et al., 2011). We were not able to measure blood pressure continuously but used the average of 3 sitting and 3 standing systolic and diastolic blood pressure readings obtained during a single Clinical Research Facility assessment. Continuous monitoring would allow assessment of other factors such as blood pressure variability on progression of WMH volume.

Notwithstanding these limitations, we have shown that, while blood pressure may be the most important determinant of WMH progression in midlife (Debette et al., 2011), lower HDL cholesterol and current smoking were the most important VRF determinants for increased rates of WMH progression from 73 years to 76 years of age. This lends support to smoking cessation and further trials of cholesterol lowering medications to reduce WMH progression in the eighth decade of life.

## Disclosure statement

Joanna M. Wardlaw reports money (grants) paid to The University of Edinburgh from Medical Research Council, Age UK, and Scottish Funding Council for her efforts on the LBC1936 study. Ian J. Deary reports money (grants) paid to The University of Edinburgh from Medical Research Council and Age UK for his efforts on the LBC1936 study. Ian J. Deary reports money paid to him for board membership on Medical Research Council. All other authors have no disclosures.

## Figures and Tables

**Table 1:** Mean ± SD volumes of whole brain and WMH at 73 y and 76 y

Structure	Mean ± SD volume at 73 y, %ICV (mL)	Mean ± SD volume at 76 y, %ICV (mL)	Mean ± SD volume change, %ICV (mL)	Cohen's *d* of change^a^	*p* value
Whole brain	69.05 ± 2.12 (994.6 ± 92.4)	67.82 ± 2.17 (975.8 ± 90.8)	−1.23 ± 1.29% (−18.8 ± 19.5)	−0.95	<0.0001
WMH	0.83 ± 0.81 (11.9 ± 11.7)	1.10 ± 1.00 (15.9 ± 14.6)	0.28 ± 0.29 (4.0 ± 4.3)	0.95	<0.0001

Key: ICV, intracranial; SD, standard deviation; WMH, white matter hyperintensities.

a Cohen's *d* were calculated using mean and SD volume change %ICV, i.e., changes in %ICV were *d* SD different to zero.

**Table 2:** Prevalence of VRF between returning and nonreturning subjects

Measure	Returners	Nonreturners	Test statistic, *p*-value
Systolic blood pressure at 73 y	146 ± 18 mmHg	146 ± 15 mmHg	*t* = 0.03, *p* = 0.975
Diastolic blood pressure at 73 y	79 ± 9 mmHg	80 ± 8 mmHg	*t* = −0.35, *p* = 0.724
HDL cholesterol ratio at 73 y	3.7 ± 1.1	3.8 ± 1.1	*t* = −0.52, *p* = 0.600
HbA1c at 73 y	5.7 ± 0.6 DCCT	5.7 ± 0.5 DCCT	*t* = 0.75, *p* = 0.451
Cardiovascular disease at 73 y	26.2%	28.7%	z = −0.55, *p* = 0.292
Hypertension at 73 y	48.2%	53.3%	z = −1.00, *p* = 0.158
Hyperlipidemia at 73 y	42.2%	37.7%	z = 0.90, *p* = 0.184
Total cholesterol at 73 y	5.2 ± 1.2 mmol/L	5.1 ± 1.0 mmol/L	*t* = 0.25, *p* = 0.805
Diabetes at 73 y	9.0%	9.8%	z = −0.30, *p* = 0.382
Smoking at 73 y	6.2%	8.2%	z = −0.80, *p* = 0.212
BMI at 73 y	27.8 ± 4.3	28.1 ± 4.7	*t* = −0.68, *p* = 0.495

Key: BMI, body mass index; DCCT, Diabetes Control and Complications Trial; HbA1c, glycated hemoglobin; HDL, high-density lipoprotein; VRF, vascular risk factors.

**Table 3:** Longitudinal changes in VRF in returning subjects

Measure	Baseline (73 y)	Follow-up (76 y)	Test statistic, *p*-value
Systolic blood pressure	146 ± 18 mmHg	147 ± 19 mmHg	*t* = 0.71, *p* = 0.480
Diastolic blood pressure	79 ± 9 mmHg	80 ± 10 mmHg	*t* = 1.81, *p* = 0.072
HDL cholesterol ratio	3.7 ± 1.1	3.7 ± 1.0	*t* = −1.23, *p* = 0.220
HbA1c	5.7 ± 0.6 DCCT	5.9 ± 0.7 DCCT	*t* = 4.57, *p* < 0.001^∗^
Cardiovascular disease	26.2%	32.4%	z = 2.08, *p* = 0.019^∗^
Hypertension	48.2%	54.9%	z = 2.06, *p* = 0.020^∗^
Hyperlipidemia	42.2%	48.3%	z = 1.86, *p* = 0.031^∗^
Total cholesterol	5.2 ± 1.2 mmol/L	5.0 ± 1.2 mmol/L	*t* = −4.68, *p* < 0.001^∗^
Diabetes	9.0%	11.9%	z = 1.49, *p* = 0.068
Smoking	6.1%	6.0%	z = −0.13, *p* = 0.449
BMI	27.8 ± 4.3	28.3 ± 14.9	*t* = 0.85, *p* = 0.395

Key: BMI, body mass index; DCCT, Diabetes Control and Complications Trial; ​HbA1c, glycated hemoglobin; HDL, high-density lipoprotein; VRF, vascular risk factors.

^∗^*p* < 0.05.

**Table 4:** Spearman correlation matrix of VRF at 73 y and WMH volume at 73 y and 76 y

Variable	DBP	SBP	HBP	HDLR	TCHL	HCHOL	HbA1c	DIAB	EXSMOK	SMOK	WMH73	WMH76
DBP												
SBP	0.569 (<0.001)^∗^											
HBP	−0.058 (0.233)	0.002 (0.962)										
HDLR	0.054 (0.265)	0.050 (0.302)	−0.069 (0.155)									
TCHL	0.150 (0.002)^∗^	0.138 (0.004)^∗^	−0.255 (<0.001)^∗^	0.444 (<0.001)^∗^								
HCHOL	−0.129 (0.008)^∗^	−0.030 (0.542)	0.333 (<0.001)^∗^	−0.173 (<0.001)^∗^	−0.187 (<0.001)^∗^							
HbA1c	−0.039 (0.422)	−0.009 (0.855)	0.138 (0.004)^∗^	0.065 (0.180)	−0.113 (0.019)^∗^	0.123 (0.011)^∗^						
DIAB	−0.069 (0.156)	−0.041 (0.399)	0.147 (0.002)^∗^	−0.082 (0.089)	−0.286 (<0.001)^∗^	0.180 (<0.001)^∗^	0.461 (<0.001)^∗^					
EXSMOK	0.002 (0.959)	0.073 (0.130)	−0.008 (0.867)	0.009 (0.852)	−0.111 (0.022)^∗^	0.090 (0.062)	0.041 (0.402)	0.079 (0.104)				
SMOK	−0.031 (0.528)	−0.031 (0.526)	0.021 (0.668)	0.047 (0.329)	0.051 (0.290)	−0.055 (0.257)	0.054 (0.262)	−0.020 (0.677)	−0.229 (<0.001)^∗^			
WMH73	0.122 (0.012)^∗^	0.137 (0.005)^∗^	0.072 (0.135)	−0.171 (<0.001)^∗^	−0.018 (0.710)	0.082 (0.090)	−0.077 (0.111)	−0.018 (0.718)	−0.012 (0.800)	0.045 (0.353)		
WMH76	0.120 (0.013)^∗^	0.126 (0.009)^∗^	0.074 (0.124)	−0.178 (<0.001)^∗^	−0.028 (0.557)	0.082 (0.089)	−0.054 (0.263)	−0.003 (0.949)	−0.022 (0.643)	0.058 (0.234)	0.979 (<0.001)^∗^	

Key: DBP, diastolic blood pressure at 73 y; DIAB, diagnosis of diabetes at 73 y; EXSMOK, ex-smoker at 73 y; HbA1c, glycated hemoglobin at 73 y; HBP, diagnosis of high blood pressure at 73 y; HCHOL, diagnosis of hyperlipidemia at 73 y; HDLR, high-density lipoprotein cholesterol ratio at 73 y; SBP, systolic blood pressure at 73 y; SMOK, smoker at 73 y; TCHL, total cholesterol at 73 y; VRF, vascular risk factors; WMH73, white matter hyperintensity volume at 73 y; WMH76, white matter hyperintensity volume at 76 y.

^∗^*p* < 0.05.

**Table 5:** Generalized estimating equations for VRF associations with change in WMH volume from 73 y to 76 y

Parameter	Beta	Standard error	Z	*p* value
ICV	0.0191 mL	0.0065 mL	2.95	0.0032^∗^
Age at baseline MRI	0.0002 %ICV	0.0002 %ICV	1.28	0.2014
0.0027 mL	0.0021 mL	1.28	0.2
Time point (baseline vs. follow-up)	0.0915 %ICV	0.0047 %ICV	19.41	<0.0001^∗^
1.3208 mL	0.0696 mL	18.98	<0.0001^∗^
Systolic blood pressure at 73 y	0.0016 %ICV	0.0029 %ICV	0.57	0.5695
0.0271 mL	0.0391 mL	0.69	0.4878
Diastolic blood pressure at 73 y	0.0076 %ICV	0.0062 %ICV	1.23	0.2196
0.08 mL	0.0824 mL	0.97	0.3319
Hypertension diagnosis at 73 y	0.0634 %ICV	0.0979 %ICV	0.65	0.5169
1.128 mL	1.31 mL	0.86	0.3892
HDL ratio at 73 y	−0.1451 %ICV	0.0531 %ICV	−2.73	0.0063^∗^
−1.798 mL	0.7754 mL	−2.32	0.0204^∗^
Total cholesterol at 73 y	−0.002 %ICV	0.0573 %ICV	−0.03	0.9725
−0.1366 mL	0.8266 mL	−0.17	0.8687
Hyperlipidemia diagnosis at 73 y	0.178 %ICV	0.0966 %ICV	1.84	0.0654
2.1952 mL	1.2761 mL	1.72	0.0854
HbA1c at 73 y	−0.1978 %ICV	0.1154 %ICV	−1.71	0.0866
−2.6515 mL	1.5789 mL	−1.68	0.0931
Current smoker at 73 y	0.4258 %ICV	0.1924 %ICV	2.21	0.0269^∗^
5.4915 mL	2.5724 mL	2.13	0.0328^∗^
Ex-smoker at 73 y	0.0383 %ICV	0.0934 %ICV	0.41	0.6817
0.6345 mL	1.2614 mL	0.5	0.6149
Diabetes diagnosis at 73 y	0.215 %ICV	0.2459 %ICV	0.87	0.3818
3.1418 mL	3.3369 mL	0.94	0.3464
Gender	0.1217 %ICV	0.0925 %ICV	1.31	0.1886
3.4228 mL	1.6715 mL	2.05	0.0406^∗^
CVD diagnosis at 73 y	−0.0539 %ICV	0.117 %ICV	−0.46	0.6448
−1.3817 mL	1.6171 mL	−0.85	0.3929
BMI at 73 y	0.0164 %ICV	0.0134 %ICV	1.23	0.2204
0.2322 mL	0.1809 mL	1.28	0.1994

There are separate models for WMH volume divided by ICV (indicated by “%ICV”) and WMH volume in mL with ICV added as a covariate (indicated by “mL”).

Key: BMI, body mass index; CVD, cardiovascular disease; HbA1c73, glycated hemoglobin; HDL, high-density lipoprotein; ICV, intracranial volume; MRI, magnetic resonance imaging; VRF, vascular risk factors; WMH, white matter hyperintensities.

^∗^*p* < 0.05.
